# Evaluating the Activity of Pigs with Radio-Frequency Identification and Virtual Walking Distances

**DOI:** 10.3390/ani13193112

**Published:** 2023-10-06

**Authors:** Anita Kapun, Felix Adrion, Eva Gallmann

**Affiliations:** Institute of Agricultural Engineering, University of Hohenheim, Garbenstraße 9, 70599 Stuttgart, Germany; fadrion@outlook.de (F.A.); eva.gallmann@uni-hohenheim.de (E.G.)

**Keywords:** UHF-RFID, activity measure, walking activity, fattening pigs

## Abstract

**Simple Summary:**

The greater the number of animals, the harder it becomes to monitor the health status of individual animals. The purpose of this study was to calculate a measure of activity for individual pigs in a fattening barn by using an automatic detection system. The results show changes in the activity data of group-housed pigs during fattening periods. We assume a high potential to use this automatically calculated activity measure for various purposes.

**Abstract:**

Monitoring the activity of animals can help with assessing their health status. We monitored the walking activity of fattening pigs using a UHF-RFID system. Four hundred fattening pigs with UHF-RFID ear tags were recorded by RFID antennas at the troughs, playing devices and drinkers during the fattening period. A minimum walking distance, or virtual walking distance, was determined for each pig per day by calculating the distances between two consecutive reading areas. This automatically calculated value was used as an activity measure and not only showed differences between the pigs but also between different fattening stages. The longer the fattening periods lasted, the less walking activity was detected. The virtual walking distance ranged between 281 m on average in the first fattening stage and about 141 m in the last fattening stage in a restricted environment. The findings are similar to other studies considering walking distances of fattening pigs, but are far less labor-intensive and time-consuming than direct observations.

## 1. Introduction

Health and welfare in pigs are important, not only for the well-being and protection of individuals, but they can also impact system profitability and sustainability [1]. Matthews et al. [1] claimed that one can utilize behavioral changes to achieve an early detection of welfare compromises. Animals’ activity patterns could be related to their reproductive cycle, social interactions, welfare, illness or lameness [1,2,3,4,5]. The monitoring of individual animals can help with detecting health issues at an early stage. Farmers can profit from support through technical assistance.

The activity level of animals has the potential to represent behavioral patterns relevant to lameness detection [6]. Lameness not only impairs welfare, but also has negative consequences for the farmer’s economic efficiency [7]. Grégoire et al. [8] discovered that lameness in sows results in less time spent in a standing position and affects the behavior during that time, especially with an increase in stepping behavior during feeding, which might be caused from discomfort when putting their weight on an affected leg. These findings are consistent with observations in some other studies with different animals, such as dairy cows [9,10,11]. Lameness often also leads to slower walking, less time spent walking, shorter distances walked and shorter stride lengths [7,8,12,13,14].

The walking behavior of pigs is influenced by many different factors. Wild boars are known to be able to walk several kilometers per day. Podgórski et al. [15] found daily distances traveled by wild boars between 2.5 and 26.7 km. The variety in these findings results from different factors, such as habitat structure, human pressure, season, weather, age, daytime and light conditions [15,16,17]. Gustafsson et al. [18] compared the foraging strategies of domestic and crossbred pigs (Holland Landrace × Wild boar) and found that the latter spent a shorter time in one place and more energy to obtain a similar amount of food to the domestic pigs by foraging a larger area and crossing more barriers. They hypothesize that domestic pigs use less costly strategies in their behavior [18]. Not much is known at present about how much distance group-housed pigs cover per day [19]. This is mainly due to the fact that accelerometers usually cannot be fixed to the animals directly with a collar around the neck or to the leg in a production environment, as they would be chewed on [6,20,21]. With small accelerometers, the application in ear tags for pigs is possible and has been used in different studies [6,12,22,23] but not for measuring walking distance itself. Distances walked are usually recorded manually, which is very cost-intensive and time-consuming [20,24]. The use of positioning sensors [6,12] and image analysis is also possible [25].

Schenck et al. [24] investigated the effects of exercising stall-housed gestating gilts and observed the distance walked of group-housed sows with different stocking densities manually for about 4 h. The results ranged between 136 and 258 m per sow for the most active period of the day, and it was concluded that the distance walked daily would be two to three times higher (272–516 to 408–774 m) [24]. Junge [19] recorded walked distances of group-housed gestating sows manually during 2 h periods and automatically with the help of a low-frequency RFID (radio-frequency identification) system, and calculated the distances between two registered reading points. The results of the direct observation for 2 h ranged between 0 and 363 m walked [19]. The calculated minimal walking distances ranged between 0 and 1712 m per sow and day with a mean value at 171 m [19].

Not many published results on the distance walked per day could be found for group-housed fattening pigs. Brendle and Hoy [20] measured the distances covered by fattening pigs at the “beginning”, in the “middle” and at the “end” of the fattening period with the help of video recordings and the software VideoMotionTracker^®^ (Mangold International GmbH, Arnstorf, Germany). The results showed highly significant (*p* < 0.001) differences, with a distance of 582 m covered per 24 h on average at the beginning, 391 m in the middle and 261 m at the end of the fattening period. The use of a video tracking software requires a video recording of the whole pen and is very time-consuming due to the need to follow the movements of the single pigs manually with the computer mouse or other input devices. In the present study, an automatic calculation of a measure of activity based on walking distances and UHF-RFID (ultra-high frequency) was developed based on the data of 400 fattening pigs. 

## 2. Materials and Methods

### 2.1. Animals, Research Barn and Technical Equipment

Data were collected during four fattening periods in a conventional fattening pig barn (University of Hohenheim, Agricultural Sciences Experimental Station, Germany). The first fattening period lasted from August to December 2016, the second lasted from January to May 2017, the third fattening period was between June and October 2017 and the fourth was between July and November 2018. In each fattening period, four mixed-gender groups of 25 pigs each were tagged with UHF-RFID transponder ear tags. The pigs (German Landrace × Pietrain) came from the rearing stable at the experimental station and were chosen by the staff considering similar age and weight. 

**Figure 1:**
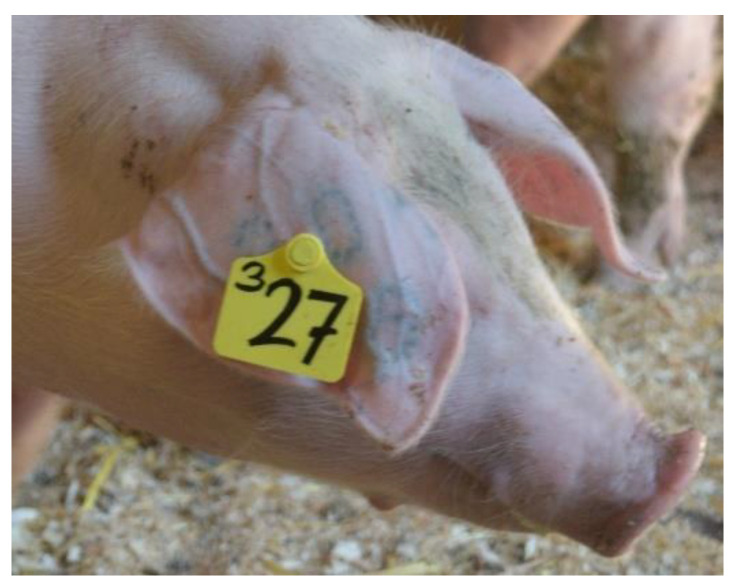
UHF-RFID transponder ear tag on a pig of about 30 kg.

The ear tags used are not commercially available, but were prototypes in another research project (Primaflex^®^, Caisley International GmbH, Bocholt, Germany) [26,27]. Figure 1 shows a pig of about 30 kg with the ear tag used. The trough, the drinkers and the playing device were equipped with UHF-RFID antennas connected to a reader (deister electronic GmbH, Barsinghausen, Germany, and Agrident GmbH, Barsinghausen, Germany) in each of the four pens, so that the pigs could be detected at those places (one potential reading per second). 

**Figure 2:**
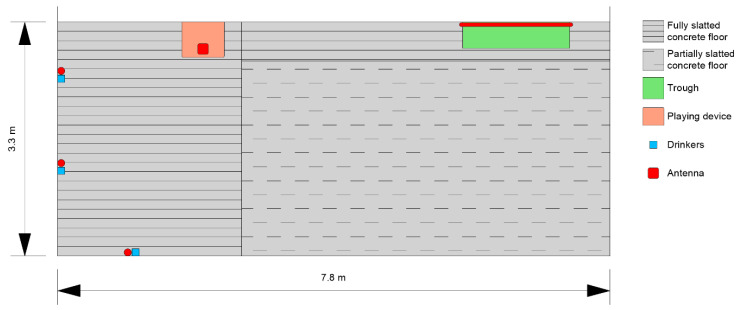
Design of one pen (created with Vectorworks 2023).

A cable antenna (Locfield^®^, Cavea Identification GmbH, Olching, Germany, 2 m active length) was installed inside a plastic pipe along the feeding trough (about 1.50 m in length). The same kind of cable antenna was set up vertically in plastic pipes next to each nipple drinker (0.35 m active length). Midrange antennas (MIRA-100, Kathrein Sachsen GmbH, Mühlau, Germany) were installed on top of the playing devices and directed vertically downwards. Figure 2 shows a pen with the locations for trough, drinkers and playing device, as well as the antenna positions. The other three pens had a similar design, with either a mirrored or the same arrangement. The pens were located in two identical compartments with forced ventilation, temperature control and slurry channels. The pigs had available space of about 1 m^2^ per pig. The antennas at the drinkers were always located to the right side of the drinkers, because of the transponder ear tags in the pigs’ right ears. A visualization of the different antennas in the pen is contained in Appendix A.

The feeding was distributed by the liquid feeding system starting at 6 a.m. There were six feeding times (two doses each), ending at 10 p.m. with the last feeding. The feeding strategy over the fattening period was a multiphase feeding program. Artificial light was provided during the same hours (from 6 a.m. until 10 p.m.) in addition to natural daylight. The playing device was a rack with straw and a wooden beam and chains (“Porky Play,” Zimmermann Stalltechnik GmbH, Eberhardzell, Germany).

The fattening periods lasted up to 19 weeks from the first day to the day the last pigs achieved the desired slaughter weight of about 120 kg. The duration of fattening was individually determined for the pigs according to their weight gain. The first pigs reached the desired weight after about 90 days of fattening (13 weeks) and were moved out earlier due to this reason. Some pigs needed to be moved to another barn earlier because of health conditions (e.g., tail biting or severe lameness). The fattening periods were divided into different fattening stages (day 1–30, 31–60, 61–90, and >90) for analyzing purposes. Each fattening stage contains data of all pigs in the trial, and therefore of all fattening periods.

The live weight of the animals was measured and recorded at the beginning of the fattening period and then again about every four weeks. The pigs had an average weight gain of 885 ± 112 g/day over all four fattening periods. The health status of the individual pigs was inspected twice a week, including lameness (locomotion scoring, LS, from 0 to 3, ZINPRO Feet First^®^, Eden Prairie, United States of America). A locomotion score of 0 is for pigs that show no signs of lameness when moving. Pigs showing visible signs of lameness but are not affected to a great extent are scored as LS 1. A score of 2 was assigned to lame pigs (one or more limbs) with additional compensatory behavior. Pigs with an LS of 3 are reluctant to walk, and try to avoid weight on one or more legs. Other health indicators, such as skin and tail lesions or soiling, were also recorded, but are not relevant for the evaluation of the results in the present work.

Table 1 shows the average starting weight of the pigs per pen in the four fattening periods as well as the mean daily temperature and relative humidity inside the two compartments.

The experimental procedures were approved by the regional authorities in Baden-Württemberg, Germany, and were carried out in accordance with EU Directive 2010/63/EU for animal experiments (approval code A 409/16 VT).

### 2.2. Virtual Walking Distance

The Euclidian distances between the center points of the antennas (two-dimensional) were used for the calculation of the distances between two antenna locations. The virtual walking distance was then calculated with the visiting order of the antenna locations and summed up for each day in Excel with a self-coded VBA macro (Visual Basic for Applications). This distance is not the distance actually walked by the pigs, but it can be seen as an activity measure. The distances between the antenna locations are listed in Table 2. A simple exemplary calculation is provided in Appendix A.

The sensitivity with which the transponders were read through the antennas was determined and has been published previously [28]. It was tested with different output powers in the first fattening period to determine the best setting for each antenna location. It was validated again for the trough in the second fattening period at different stages (beginning, middle, and end of the fattening). The sensitivity ranged between about 60% at the drinkers and up to about 90% at the trough. However, these were calculated with readings aggregated to visits. The visiting time was not important, and single readings of pigs passing by should not be excluded; therefore, the single RFID readings were used for the calculation of the virtual walking distance. Even though the pigs were not always detected when they were in the reading area of the antennas, all readings could be used for the calculation of a minimum distance walked, because a reading could not have happened if the pig was too far away from the antenna. However, due to the horizontal expanse of the reading area of an antenna and the distinction to the center point, there is a very small possibility that the calculation could also be too high. Mainly for this reason, this activity measure was called the virtual walking distance instead of the minimum walking distance.

## 3. Results

**Figure 3:**
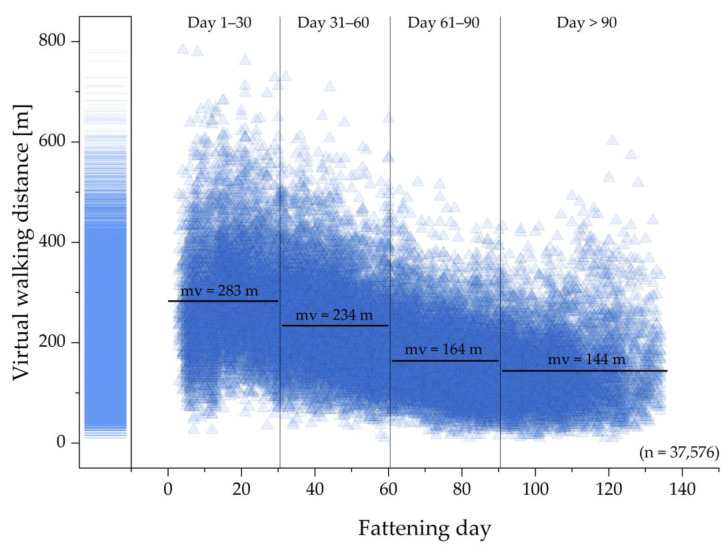
Daily virtual walking distances of all 400 pigs. Each data point has a transparency of 80%; thus, the distribution is partly recognizable by the density of data points.

The daily calculated virtual walking distances of all 400 pigs are illustrated over the fattening days in Figure 3. The data points’ transparency of 80% is intended to emphasize their distribution. The calculation of the virtual walking distances for the pigs of the first fattening period started at fattening day 25, because there was a validation phase for the RFID system prior to that. For the pigs of the other fattening periods, the calculation started at fattening day 4, 5 or 6, the day after they received the UHF-RFID ear tags. There are over 35,000 data points in total, and the mean value for the virtual walking distance per day was 205 m. The highest value for the virtual walking distance was almost 800 m per day, whereas the lowest value was about 10 m. The lowest value was calculated for five different pigs on different days. Those pigs were only read at the trough, the playing device and then again at the trough throughout the entire day. Technical issues can be ruled out because other pigs were detected regularly. It is possible that these pigs were at the drinkers and were not detected there. However, it is also possible that they were not at the drinkers, because the liquid feeding can cover a pig’s fluid intake need.

The virtual walking distance shows a tendency to decrease over time. The results can be divided into different fattening stages (day 1–30, 31–60, 61–90, and >90). The mean values (mv) of all daily virtual walking distances calculated are 283 m for day 1–30 (*n* = 7883), 234 m for day 31–60 (*n* = 11,084), 164 m for day 61–90 (*n* = 10,876) and 144 m for day >90 (*n* = 7733). 

**Figure 4:**
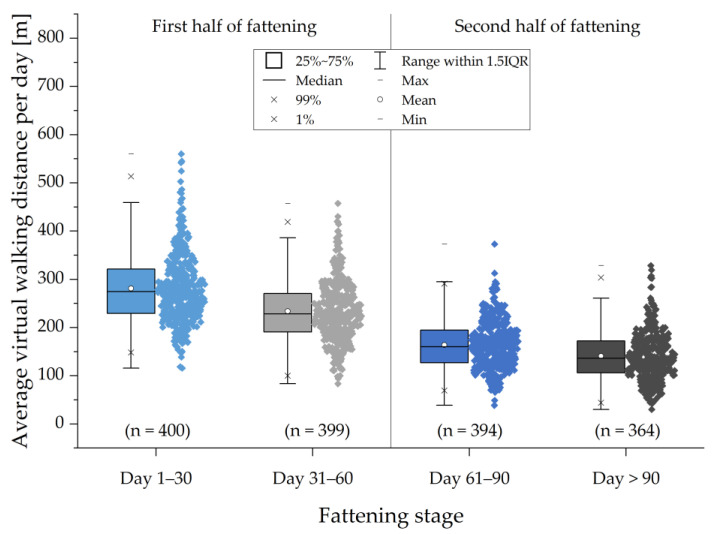
Average virtual walking distance per day of the 400 individual pigs, divided into four fattening stages (day 1–30, day 31–60, day 61–90 and day >90).

The mean values of individual pigs were used as data for Figure 4 and, again, divided into four fattening stages. Not surprisingly, the average values per pig are very similar to the complete dataset with 281 m for day 1–30 (*n* = 400), 234 m for day 31–60 (*n* = 399), 164 m for day 61–90 (*n* = 394) and 141 m for day >90 (*n* = 364). The values once again show a decrease from the beginning to the end of the fattening periods. The data points are less scattered and high or very low values have a great informative content because the individual pigs would need to have these high or low values for a certain time. The highest value for the average virtual walking distance of a single pig during one of the fattening stages is 560 m (pig 386, day 1–30), whereas the lowest value is at about 30 m (pig 188, day >90).

**Figure 5:**
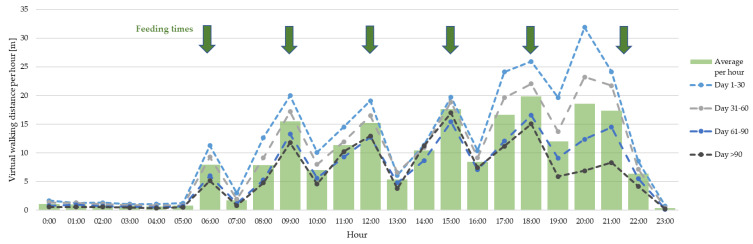
Average virtual walking distance of all 400 pigs during the hours of the day, divided into four fattening stages (day 1–30, day 31–60, day 61–90 and day >90) and average over all fattening days (green columns). The feeding times are indicated with green arrows.

Figure 5 shows the average virtual walking distance of all 400 pigs, divided into the different hours of a day as an average value and divided into the same four fattening stages as before. The data at 0:00, for example, state the calculated walking distances at the time between 12 a.m. and 1 a.m., and so forth. The data suggest that the pigs are walking less at night between 10 p.m. and 6 a.m., and are presumably using that time as a resting period. The course of the graph is similar for all fattening stages, only the level changes and decreases most of the time. The feeding times seem to have an influence on the walking activity with lower values between feedings. Overall, the time in the afternoon shows a higher activity, but the hours after feeding seem to stand out as times where the pigs were less active. The highest average walking distance per hour was calculated for the time between 8 p.m. and 9 p.m. in the first fattening stage (day 1–30) and amounts to 31.9 m. This varies for the four different fattening stages. The hour with the highest activity, on overall average, is the time between 6 p.m. and 7 p.m. (19.8 m). 

**Figure 6:**
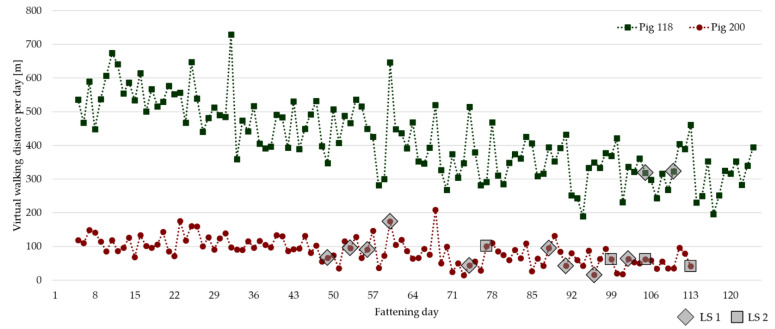
Virtual walking distance of the pig with the lowest average value (pig 200, about 87 m per day) and the pig with the highest average value (pig 118, about 416 m per day). Lameness observations are marked with grey squares (rhombic squares for LS 1 and squares for LS 2).

The virtual walking distances calculated of all 400 pigs range between an average of 87 and 416 m per day (per pig). There is not only a high inter-variability between different animals, but also a high intra-variability, which means a high variability in the data of single pigs on different days. Figure 6 displays the virtual walking distance per day of the pig with the lowest average value (pig 200) and the pig with the highest average value (pig 118) during the entire duration of the study. Both were in the same fattening period in different pens and left the trial on the same day for slaughter, but there are no data for the last few days for pig 200 due to technical issues in pens 3 and 4.

The lameness detection for pig 118 showed slightly visible signs of lameness (LS 1) on two consecutive health observation days (fattening days 105 and 110) and no signs of lameness before or after that. Pig 200 had more observation days with a locomotion score of 1 (11 in total, day 116 and 119 not shown in the figure), and even showed lameness in one or more limbs (LS 2) on fattening day 77, and on four out of eight health observation days between fattening day 99 and the last day of fattening (last observation on day 123 not shown in the figure). Both pigs show the tendency to reduce their walking activity in the course of the fattening period.

**Figure 7:**
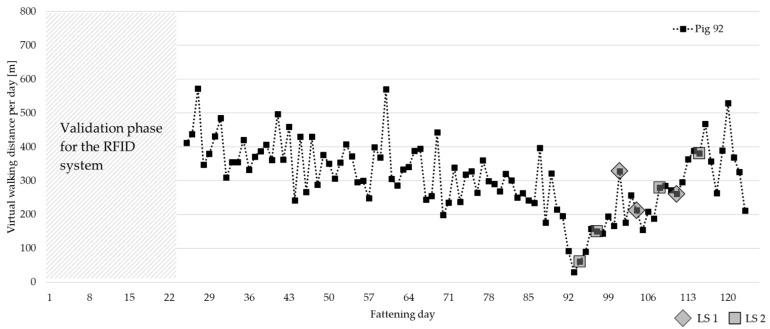
Virtual walking distance per day of pig 92 with first signs of lameness on fattening day 94 (calculation starts on fattening day 25 due to the system’s validation phase in the first weeks of fattening). Lameness observations are marked with grey squares (rhombic squares for LS 1 and squares for LS 2).

Figure 7 shows an example where the virtual walking distance of a pig could be related to their health observation. There are no values for the first 24 days due to the system’s validation phase in the first weeks of fattening period 1. Pig 92 showed the first sign of lameness on fattening day 94 with a locomotion scoring of 2. The activity measure virtual walking distance per day drops clearly around the same time. Lameness was recorded for that pig from fattening day 94 until fattening day 115 (between LS 1 and LS 2 on seven health observation days). The virtual walking distance rises again after a few days to the same level as before without the lameness being completely gone.

## 4. Discussion

The present study shows the possibility of using a simple RFID system to assess the walking activity of animals, in this case, fattening pigs. The results show a high variability, but are comparable to studies with direct observation and calculation of distances covered by pigs. The results for average distances walked per day range between 87 and 416 m for single pigs. Divided into different stages of fattening, the mean values range between 281 m (day 1–30), 234 m (day 31–60), 164 m (day 61–90) and 141 m (day >90). Brendle and Hoy [20] investigated the distance covered by fattening pigs per day by using a software tool and manually tracking the pigs with a PC mouse or another device and found similar results. They evaluated the daily walking distances at three different times throughout the fattening period (“beginning,” “middle” and “end”) for two days each. These three fattening stages are roughly comparable to the first three fattening stages in the present study until fattening day 90. Brendle and Hoy found daily walking distances of about 582 m at the “beginning,” 391 m in the “middle” and about 261 m at the “end” of the fattening period [20]. These values are more precise due to the direct observation, and thus higher than the virtual walking distances we found, but they also show a decrease over time. Differences in walking activities may also occur due to different group or housing sizes, as well as between female and castrated male pigs [20]. Other influencing factors are the layout of the pens and whether the pigs have access to an outdoor area.

Considering the results for the virtual walking distances during the different hours of the day, a resting period during the night can be concluded (from about 11 p.m. until 6 a.m.). This coincides with the time the feeding system was not running at night. Using the walking values as an activity measure, the pigs became more active toward the afternoon and evening (from about 5 p.m. until 8 p.m.). This agrees with results from many other studies on the behavior of pigs, for example, a study on the behavior of domestic pigs under near-natural conditions [29]. However, a biphasic activity and feed intake rhythm with a quieter phase at noon, as it is commonly reported in various textbooks, could not be detected in this study or in our study [30]. In the present setup, one reason for a more frequent activity with shorter break times could be the feeding system with six feeding times (two doses each, about every three hours) during the day. The activity in pens with ad libitum feeding in an older study at the same pig barn showed an approximately biphasic development, whereas the activity in pens with liquid feeding (twenty feeding times during the day) seemed to be determined by the feeding times [31]. 

A permanent monitoring of the general walking activity of single pigs can be achieved in the present experimental setup with relatively little effort. It is less time- and cost-intensive than direct observations or video analysis by hand. The detection rate might be considered challenging, but since the sensitivity was calculated by comparing the duration of stay and a considerably continuous detection of the RFID ear tags, it is concluded to be sufficient for the walking distance calculation most of the time. A single detection is enough to count into the calculation of virtual walking distance, and it is presumed that these detections are less likely to be missed the longer the visits are. In comparison to automatic image analysis for tracking pigs, the present approach with a UHF-RFID system can deliver reliable data for individual animals. Regarding computer vision, it is difficult to distinguish the individual pigs without markings on the animals, be they natural colorations of the skin or manually marked [32]. Image analysis, however, has the advantage of potentially detecting a larger variety of behavioral activities by observing, for example, posture, position or lying patterns [33]. A combination of both computer vision and RFID for identification might be the most promising approach to automatically detect important behavioral patterns for pigs, or animals in general, on an individual level. 

Lameness detection might become possible by using these virtual walking distances, as seen in the present results, but not every lameness is clearly visible in the daily walking data. Further research, possibly with assistance from artificial intelligence technologies, is needed.

The virtual walking distance is a measure of activity and does not reflect the actual distance walked by pigs. It is to be expected that the actual distances walked are higher than the values calculated [19], but the difference is not assessable. The area not covered by the UHF-RFID system needs to be reduced to a minimum, but without overlapping reading area, to increase the comparability between these two values. The application of this system in different housing situations and larger pens with more structured functional areas could be very interesting for future studies.

Activity measures should be considered on an individual level for each animal. Different pigs showed different “base values” or “default values” in activity, which should be included into any analysis as baselines. Further research should include ways to use this automatically detected activity measure for more input on behavioral and welfare data of pigs, not only fattening pigs, and might also examine its use for lameness detection or the detection of other health and welfare impairments.

## 5. Conclusions

In the present study, an RFID system was used to automatically assess an individual activity measure by estimating minimum distances walked by animals, in this case, fattening pigs. This measure can be used to detect changes in walking activity over time or between different animals or groups. The pigs in this study tended to walk less over time, which coincides with findings of other studies on the walking activity of fattening pigs. 

## Figures and Tables

**Table 1:** Average weight of the pigs at the beginning of each fattening period and the average daily temperature and humidity during the fattening periods (mean value ± standard deviation). Pen 1 and pen 2 are in compartment C1; the other two pens are in compartment C2.

Fattening Period	Pen	Average Starting Weight of the Pigs (kg)	Compartment	Average Daily Temperature (°C)	Average Daily Relative Humidity (%)
Fattening period 1 (Aug–Dec 16)	Pen 1	32.5 ± 2.9	C1	20.0 ± 4.3	55.8 ± 6.1
	Pen 2	27.5 ± 2.5			
	Pen 3	32.6 ± 2.3	C2	20.1 ± 4.2	54.0 ± 6.4
	Pen 4	27.3 ± 1.5			
Fattening period 2 (Jan–May 17)	Pen 1	31.9 ± 3.9	C1	18.7 ± 1.6	47.7 ± 7.6
	Pen 2	36.0 ± 3.1			
	Pen 3	33.1 ± 3.4	C2	18.4 ± 1.4	46.3 ± 8.6
	Pen 4	31.7 ± 3.1			
Fattening period 3 (Jun–Oct 17)	Pen 1	29.2 ± 1.9	C1	22.6 ± 3.8	57.7 ± 7.5
	Pen 2	31.1 ± 2.8			
	Pen 3	27.6 ± 1.7	C2	22.1 ± 3.7	57.4 ± 6.3
	Pen 4	32.6 ± 2.5			
Fattening period 4 (Jul–Nov 18)	Pen 1	27.3 ± 3.1	C1	21.1 ± 4.3	52.5 ± 6.0
	Pen 2	27.0 ± 2.5			
	Pen 3	34.3 ± 2.9	C2	21.6 ± 4.4	54.7 ± 6.6
	Pen 4	25.2 ± 1.8			

**Table 2:** Euclidian distances between the center points of the five antenna locations at the trough, drinkers and playing device.

	Trough	Drinker 1	Drinker 2	Drinker 3	Playing Device
Trough	–	7.0 m	7.5 m	7.3 m	5.2 m
Drinker 1		–	1.6 m	2.7 m	3.0 m
Drinker 2			–	1.3 m	2.6 m
Drinker 3				–	2.1 m
Playing Device					–

**Figure A1:**
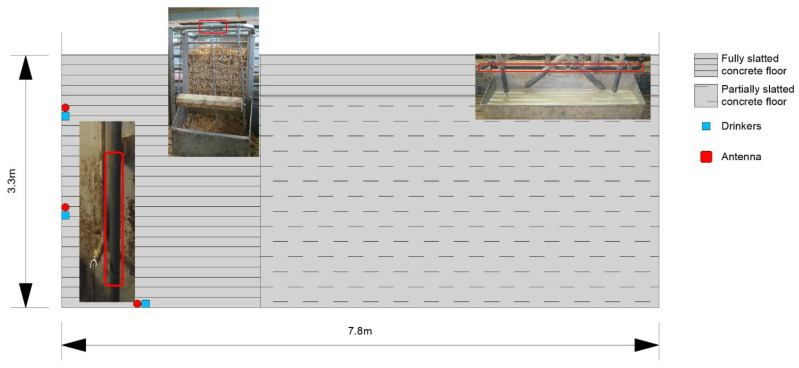
Design of one pen (created with Vectorworks 2023) with additional pictures of the different antennas.

**Figure A2:**
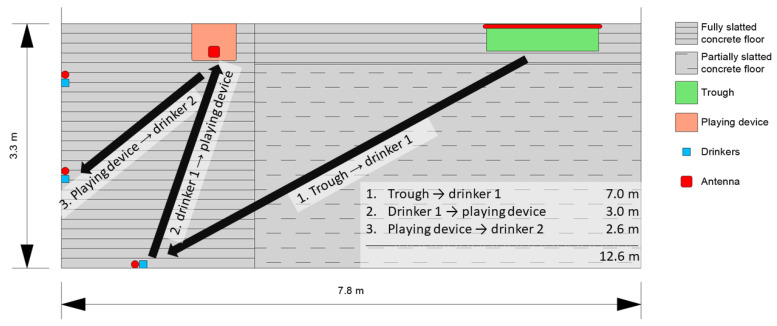
Exemplary calculation for the walking distance with detected readings at four different antennas. The pig’s visiting at the trough, drinker 1, the playing device and then drinker 2 sum up to a virtual walking distance of 12.6 m.

## Data Availability

The data presented in this study are available on reasonable request from the corresponding author. The data are not publicly available at this time as the data also forms part of an ongoing PhD thesis.
